# Supplementary material for: Adipocyte Utx Deficiency Promotes High-Fat Diet-Induced Metabolic Dysfunction in Mice
Source: Cells. 2022 Jan 6;11(2):181. doi: 10.3390/cells11020181 (PMC8773702; doi:10.3390/cells11020181)
Supplement: Supplementary file 1 [file cells-11-00181-s001.zip › cells-1507429-supplementary.pdf]

Supplementary Materials

Supplemental Figure S1. *Utx* mRNA expression in inguinal white adipose tissue (iWAT), epididymal WAT (eWAT) and interscapular brown adipose tissue (iBAT) of male AUTXKO and *fl/Y* animals. All data are presented as mean ± S.E.M.; n=4/group; *p<0.05 vs. *fl/Y*.

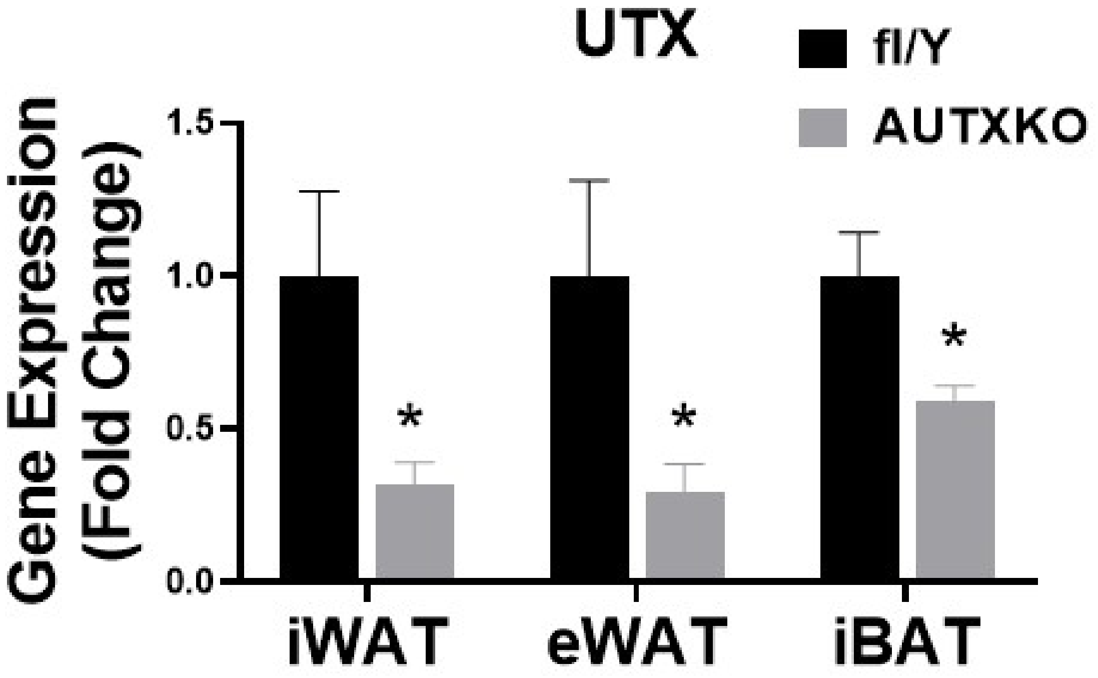

Supplemental Figure S2. *Utx* deficiency does not change mitochondrial respiration chain complex proteins in iWAT of AUTXKO mice fed chow diet. Western blots of UCP1, phosphor-HSL, total HSL, and mitochondrial respiratory chain complex proteins in the iWAT of male AUTXKO and *fl/Y* animals (on the left panel) and quantitation of blot densitometry (on the right panel). iBAT sample highlighted in red box serves as a positive control. All data are presented as mean ± S.E.M.; n=3-4/group.

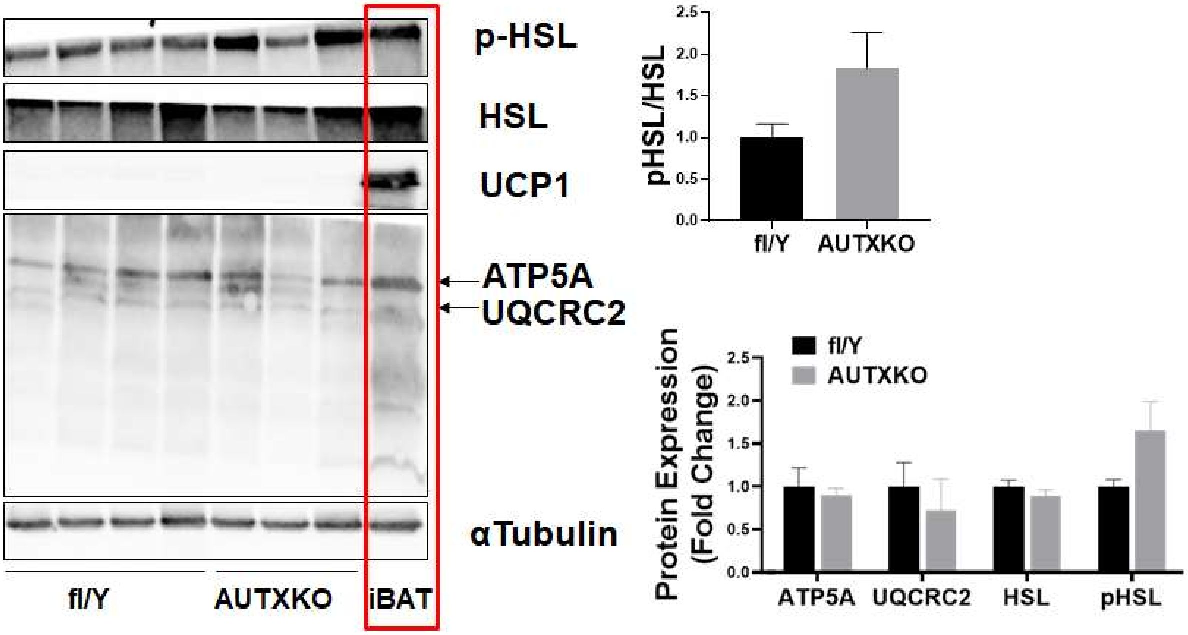

Supplemental Figure S3. *Utx* deficiency in adipocytes slightly decreases mitochondrial respiration chain complex proteins in iBAT of AUTXKO mice fed HFD. Western blots of UCP1, phosphor-HSL, total HSL, and mitochondrial respiratory chain complex proteins in the iBAT of male AUTXKO and *fl/Y* animals (on the left panel) and quantitation of blot densitometry (on the right panel). All data are presented as mean ± S.E.M.; n=7-8/group; *p<0.05 vs. *fl/Y*.

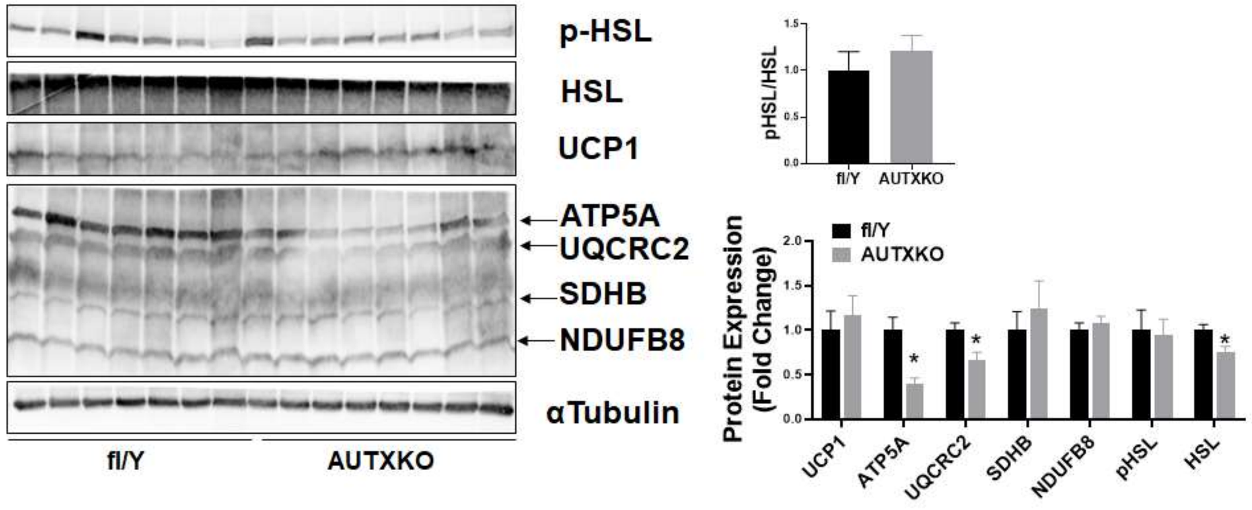

Supplemental Figure S4. *Utx* deficiency does not change mitochondrial respiration chain complex proteins in iWAT of AUTXKO mice fed HFD. Western blots of UCP1, phosphor-HSL, total HSL, and mitochondrial respiratory chain complex proteins in the iWAT of male AUTXKO and *fl/Y* mice (on the left panel) and quantitation of blot densitometry (on the right panel). iBAT sample highlighted in red box serves as a positive control. All data are expressed as mean ±

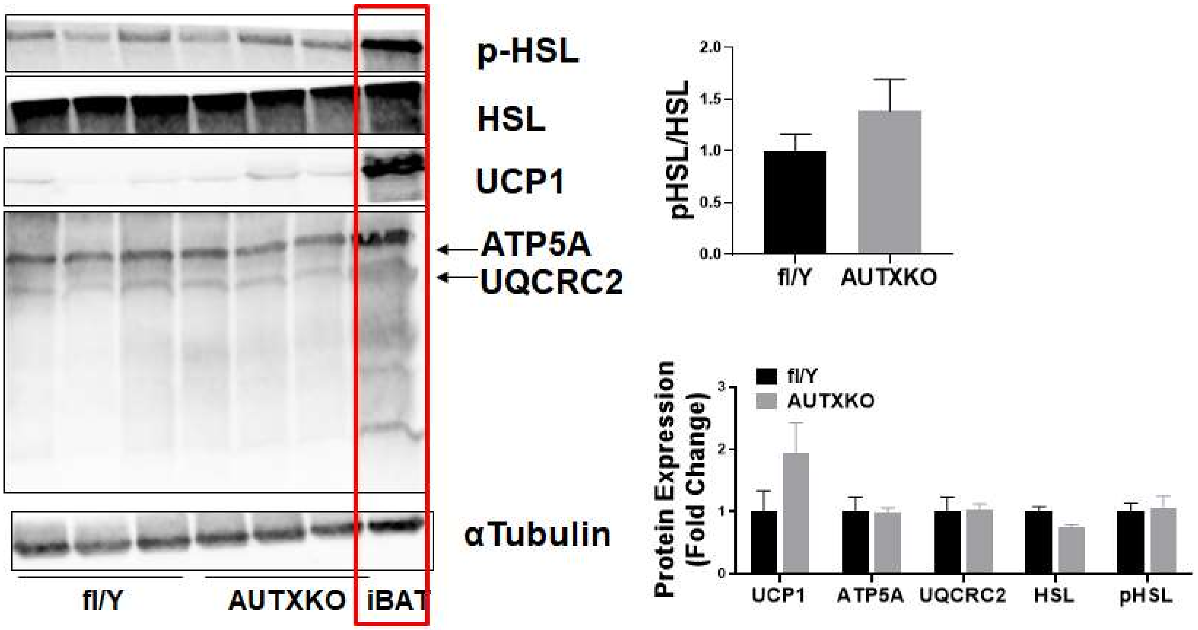

S.E.M.; n=3/group.
